# The Development of an Intelligent Agent to Detect and Non-Invasively Characterize Lung Lesions on CT Scans: Ready for the “Real World”?

**DOI:** 10.3390/cancers15020357

**Published:** 2023-01-05

**Authors:** Martina Sollini, Margarita Kirienko, Noemi Gozzi, Alessandro Bruno, Chiara Torrisi, Luca Balzarini, Emanuele Voulaz, Marco Alloisio, Arturo Chiti

**Affiliations:** 1 Department of Biomedical Sciences, Humanitas University, Via Rita Levi Montalcini 4, Pieve Emanuele, 20090 Milan, Italy; 2 IRCCS Humanitas Research Hospital, Via Manzoni 56, Rozzano, 20089 Milan, Italy; 3 Fondazione IRCCS Istituto Nazionale Tumori, Via G. Venezian 1, 20133 Milan, Italy; 4 Laboratory for Neuroengineering, Department of Health Sciences and Technology, Institute for Robotics and Intelligent Systems, ETH Zurich, 8092 Zurich, Switzerland

**Keywords:** CT scans, lung nodules, artificial intelligence, deep learning

## Abstract

**Simple Summary:**

An “intelligent agent” based on deep learning solutions is proposed to detect and non-invasively characterize lung lesions on computed tomography (CT) scans. Our retrospective study aimed to assess the effectiveness of Retina U-Net and the convolutional neural network for computer-aided detection (CADe) and computer-aided diagnosis (CADx) purposes. CADe and CADx were trained, validated, and tested on the publicly available LUNA challenge dataset and two local low-dose CT datasets from the IRCCS Humanitas Research Hospital.

**Abstract:**

(1) Background: Once lung lesions are identified on CT scans, they must be characterized by assessing the risk of malignancy. Despite the promising performance of computer-aided systems, some limitations related to the study design and technical issues undermine these tools’ efficiency; an “intelligent agent” to detect and non-invasively characterize lung lesions on CT scans is proposed. (2) Methods: Two main modules tackled the detection of lung nodules on CT scans and the diagnosis of each nodule into benign and malignant categories. Computer-aided detection (CADe) and computer aided-diagnosis (CADx) modules relied on deep learning techniques such as Retina U-Net and the convolutional neural network; (3) Results: Tests were conducted on one publicly available dataset and two local datasets featuring CT scans acquired with different devices to reveal deep learning performances in “real-world” clinical scenarios. The CADe module reached an accuracy rate of 78%, while the CADx’s accuracy, specificity, and sensitivity stand at 80%, 73%, and 85.7%, respectively; (4) Conclusions: Two different deep learning techniques have been adapted for CADe and CADx purposes in both publicly available and private CT scan datasets. Experiments have shown adequate performance in both detection and diagnosis tasks. Nevertheless, some drawbacks still characterize the supervised learning paradigm employed in networks such as CNN and Retina U-Net in real-world clinical scenarios, with CT scans from different devices with different sensors’ fingerprints and spatial resolution. Continuous reassessment of CADe and CADx’s performance is needed during their implementation in clinical practice.

## 1. Introduction

Lung lesions are common. The overall incidence of lung nodules has increased 10-fold from 1959 to 2015 [1], but–fortunately—the diagnosis of lung cancer has not risen accordingly [2]. The increasing use of “modern” imaging techniques, the higher adherence to screening programs, and the regular follow-up of patients suffering from other cancers result in a more significant number of lung lesions being incidentally detected in asymptomatic people [2]. Several factors should be considered dealing with the first diagnosis of lung nodules, including the patient’s pre-test probability of malignancy (e.g., smoking habits and familiar or previous history of lung cancer), and the lesion’s characteristics (e.g., size, spiculation, and pleura indentation) [2]. Based on these risk assessments, patients are assigned to a class of risk and are managed accordingly [2]. The workup of patients with incidentally detected pulmonary lesions comprises actions from no further steps to computed tomography (CT) surveillance, to [^18^F]FDG positron emission tomography (PET)/CT, to invasive procedures (biopsy, surgery, radiation therapy, or interventional radiology treatment). From a practical point of view, once identified, lung lesions must be characterized by assessing the risk of malignancy. Several qualitative CT features have been reported to be associated with malignancy (e.g., size and attenuation characteristics) [2,3], and standardized criteria to describe pulmonary nodules have been proposed (number, size, and pattern) [3]. Nonetheless, there are still several hurdles to be overcome concerning the applicability and reproducibility of these criteria (i.e., inter-operator and intra-operator variability due to misinterpretation and different experiences and expertise), ultimately affecting the management of patients diagnosed with lung nodule(s). 

In recent years, artificial intelligence, acting as “another pair of eyes”, has gained popularity. Computer-aided detection (CADe) and computer-aided diagnosis (CADx) systems have been recently developed [4,5,6] to support imagers in both lung lesion detection and diagnosis tasks. A number of models have been developed for the purpose of lung nodule detection and segmentation [7,8]. Many lung nodule segmentation algorithms based on either general or multiview neural network architecture have been proposed. Most studies adopting multiview neural networks have introduced new architectures by taking multiple lung nodule views. Subsequently, they use those views as inputs to the neural networks. On the contrary, the general neural-network-based methods rely primarily on U-Net architecture. Moreover, different lung nodule segmentation methods can be used for different types of lung nodules. Additionally, many techniques have been proposed for the classification of lung nodules (e.g., whether they are benign or malignant) focused on supervised, as opposed to semi-supervised, learning [7,8]. Despite the promising performance of these computer-aided systems, there are still limitations related to the study design (e.g., retrospective trial), technical issues (e.g., the manual labeling of images and high cost) and the efficiency (e.g., low calculation efficiency) of these tools. 

The study presented in this paper aimed to develop an “intelligent agent” to detect and non-invasively characterize lung lesions on CT scans. Our goal was to apply CNN for lung cancer identification on the CT scans inspired by the available literature, but more importantly we aimed to test the tool in a “real-world” setting. In greater detail, the project involved two main modules: the first one addressed the detection of lung nodules on CT scans; the second dealt with the diagnosis (CADx) of each nodule into benign and malignant categories. The “intelligent agent” relied on deep learning techniques, which are described in the following sections. 

## 2. Materials and Methods

### 2.1. Study Design

The study was a retrospective, single-institution trial. 

We used public and local datasets to develop the CADe-CADx. CADe and CADx were independently developed. The study was approved by the institutional Ethics Committee.

### 2.2. Datasets and Image Analysis 

This subsection provides details for both publicly available and local datasets for our CADe-CADx. Table 1, Table 2 and Table 3 set out lung abnormalities within the LUNA challenge dataset, CT scans used for CADs’ development, and the number of nodules used for CADx.

#### 2.2.1. LUNA Challenge Dataset

The open-source LUNA challenge dataset [9] and the local ICH_s1 and ICH_s2 datasets were used for the detection task.

The LUNA dataset consists of 805 series of diagnostic and lung cancer screening chest CT scans along with XML annotation files. Lung abnormalities have been annotated by four thoracic radiologists. Each abnormality is classified as a nodule or not, and annotated according to size, as detailed in Table 1.

The mask of the region of interest (ROI) for nodules of at least 3 mm was based on a 50% consensus criterion on four radiologists’ segmentations.

#### 2.2.2. Local Datasets—ICH_s1 and ICH_s2

ICH_s1 is a local dataset consisting of 1189 low-dose CT series. The images were independently analyzed by two expert chest radiologists, and all of the nodules were segmented on non-contrast-enhanced images regardless of size. ICH_s2 consisted of 92 annotated lesions close to the mediastinum. The “ground truth” for the CADe was the segmentation performed by imagers (full concordance between radiologists). Collectively, local datasets included 1281 CT scans (441 with at least one nodule). The above-mentioned datasets were split into three subsets (training, validation, and test), as detailed in Table 2. Therefore, test set images for both ICH_s1 and ICH_s2 were used neither for training nor validation purposes. 

The 234-test series from the ICH_s1 dataset comprises 104 nodules. One nodule per series is present in the 19-test series from the ICH_s2 dataset. Image segmentation and labelling were performed using a dedicated plug-in implemented for the 3D-slicer software tool (version 4.10.2, Slicer.org, Boston, MA, USA) [8].

#### 2.2.3. CADx—Datasets and Image Analysis 

The local datasets, ICH_x1 and ICH_x2, were used for classification tasks. The ICH_x1 subset comprised 349 low-dose CT images with nodules, with 29 confirmed to be malignant. The images were analyzed by an expert chest radiologist (CT), and all of the nodules were segmented on non-contrast-enhanced images regardless of size. There was a partial overlap between the series included in ICH_s1 and ICH_x1. The ICH_x2 subset consists of 957 CT scans (all with at least one nodule) annotated by marking the lesion centroid. ICH_x2 samples were annotated on non-contrast-enhanced images by experienced imagers (CT and MS). ICH_x2 comprises any type of CT scan acquired at our institution, including co-registered images of PET/CT (*n* = 301), biopsy-guiding CT scans (*n* = 305), and diagnostic CT scans (*n* = 351, respectively). Collectively, 1346 nodules in 1306 CT scans were segmented and labelled. Radiological follow-up and pathology were used as reference standards in 350/1346 and 996/1346 cases, respectively (Table 3). Specifically, complete resolution of lung lesions was used as a radiological reference standard to define a nodule as benign. The final radiological diagnosis was used to classify 567/632 benign nodules. In the other 65/632 cases, benign nodules were pathologically confirmed. All malignant nodules were pathologically confirmed. Malignancy included primary lung cancer (adenocarcinoma = 392/714, squamous cell carcinoma = 113/714, carcinoid tumor = 31/714, and other = 35/714) and lung metastases (*n* = 133/714). In ten patients, the primary lung tumor subtype was not specified. The final diagnosis was collected from electronic medical records. Image segmentation and labelling were performed using a dedicated plug-in implemented with the 3D slicer tool.

### 2.3. CADe and CADx Architectures

As briefly mentioned in the previous sections, deep learning paradigms are behind the proposed CADe and CADx systems. One of the main challenges in our work was to test the effectiveness of deep learning architectures in real scenarios accounting for several variables, such as different CT devices, images with different spatial resolutions, and device fingerprints.

Due to the different nature of detection and diagnosis tasks, we opted for two different deep neural network architectures. CADe relies on pixel-wise segmentation to reveal whether a pixel is part of a lung lesion. To this end, it is necessary to obtain a full-resolution output binary mask to retrieve both the coordinates and the region of the lung lesion. 

Conversely, CADx focuses on the final diagnosis of a given lung lesion. The system is meant to return a label indicating ‘benign nodule’ or ‘malignant nodule’. Then, it is not necessary to make the system to return a full-resolution output mask while only an output label is needed. The following two subsections provide further technicalities regarding the two different architectures for CADe and CADx. 

Furthermore, it is necessary to point out that deep learning networks must ingest many images to deliver a model with knowledge inference and generalization that can accomplish a specific domain task. The biomedical image analysis scenario is afflicted by a dimensionality problem due to the lack of manually annotated data. To be more accurate, the dimensionality issue refers to the size of hand-labelled data, which is not reasonably big enough to have a deep neural network trained from scratch. 

That is where data augmentation comes into play; applying image transformations without altering the meaningful content of the image itself makes a given dataset bigger in size by generating new samples. Examples of primary data augmentation are the following: flipping, mirroring, rotation, translation, and scaling. 

In the following two subsections, a further description of the deep learning techniques for CADe and CADx tasks is given.

#### 2.3.1. CADe Architecture and Development

The main goal of a CADe system is to return a full-resolution mask highlighting the suggested regions of interest for a given input image. That is why we opted for the fully convolutional neural network (FCNN) architecture. CADe tasks are, therefore, accomplished in a pixel-wise manner to extract information related to both the ROI (region of interest) and the corresponding targets. FCNN allows for return of a full-resolution mask for a given input image. In simpler terms, an FCNN ingests an input image with size M × N and returns an output mask with the exact dimensions. The latter makes it suitable for critical biomedical image analysis tasks, such as segmentation and detection. 

One of the most popular and cited FCNNs for biomedical image segmentation is the so-called U-Net [10] which owes its name to the U-shape of the network architecture. In this section, we provide readers with the overall description of U-Net, including the main layers and operations throughout the network. For the sake of clarity, we do not address the most complex mathematical concepts, and instead point the readers toward to the reference articles for further details [10].

**Figure 1:**
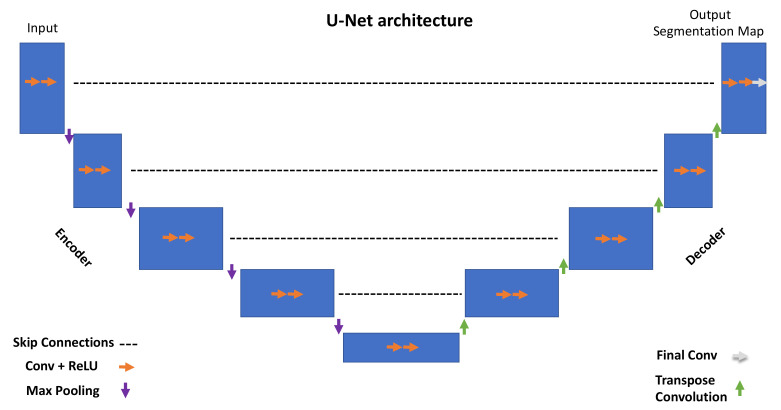
U-Net architecture.

The overall U-Net architecture is depicted in Figure 1. The encoder is responsible for extracting hidden information within the pixel domain. The latter is achieved with a stack of filters that down-sample input images in the first place. In simpler terms, the network architecture is organized in levels, with each level consisting of two Conv (convolutional layers) followed by a ReLU (rectified linear unit), a max pooling layer characterized by a parameter, namely ‘stride’, tuning the down-sampling factor for the input image. 

All of the encoder levels are meant to extract the most meaningful features from the input images all the way to the network bottom level. Each level returns outputs through feature maps (or channels). They represent intermediate stages of the network layers that feed the following level in the stack. From a graphical viewpoint, blue rectangles indicate the input, feature maps, and output of the network. Going through consecutive layers through the encoder, it is noticeable how rectangles change in size, turning into shorter but wider blue rectangles. This is a descriptive representation showing what happens inside the network: convolutional layers work as image feature extractors; ReLU is an activation function whose primary role is to give neural networks non-linearity representation capabilities to represent results with more accuracy. Max pooling is a “pooling” operator extracting the max value from image patches and bringing down down-sampled patches. 

Purple downward arrows in Figure 1 show max pooling coming into play, while orange arrows represent the sequence Conv + ReLU. The encoder is responsible for extracting “what” is in the images, while the decoder deals with the “where”.

The features extracted by the contracting path are then progressively reconstructed by the expanding path (decoder) with layers consisting of transpose convolution (deconvolution), Conv + ReLU and Final Conv. Transpose convolution allows upsampling of the feature maps out of the previous layers; Conv + ReLU are then applied in combination with skip connections to refine the results in each level. Skip connections help to retrieve missing information from the encoder feature maps standing on the same level. The top left corner of the network returns a segmentation map by adopting a one-dimensional convolutional layer. The latter can return labels in a pixel-wise fashion.

The network employed for our CADe, namely, Retina U-Net [11], is a variant of two pre-existing networks, Retina Net [12] and U-Net [10].

#### 2.3.2. Retina U-Net

**Figure 2:**
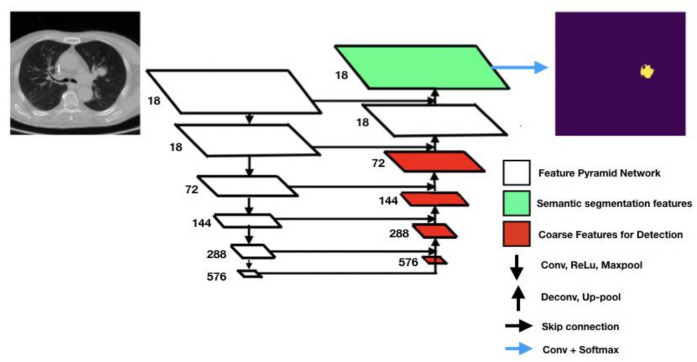
Architecture of the U-Net neural network used to segment lung nodules in CT scans. The number left on each layer represents the number of output channels.

Retina U-Net [11] integrates elements from Retina Net and U-Net to combine object detection and semantic segmentation. Taking after most of the state-of-the-art object detectors, Retina U-Net complements U-Net architecture by introducing object-level predictions through feature pyramid networks (FPNs) [13]. FPNs are feature extractors with bottom-up and top-down paths. The overall Retina U-Net architecture is graphically represented in Figure 2. The overall pipeline is mainly characterized by FPNs, coarse features detectors, skip connections, Conv + Softmax, Conv + ReLu + MaxPool. 

Coarse feature detectors, indicated by red rectangles in Figure 2, are responsible for detecting small-sized objects using sub-network operations such as the so-called bounding box regressor (a well-known object detection technique) [14]. Skip connections support the network in retrieving missing information from the encoder feature maps standing on the same level. The Conv + ReLU + MaxPool stack consists of convolutional filter, a rectified linear unit function, and a max pooling filter. They are key to the contracting path of the FCNN as Conv filters and MaxPool filters down-sample the input feature map while ReLU allows for generalization and inference of knowledge from a non-linear input (as it is a piecewise linear function).

Conv + SoftMax consists of a sequence of a convolutional filter and a SoftMax function returning a probability map for every possible class to be detected in the images. The Up-pool and Deconv layers are responsible for the image reconstruction starting from the network bottleneck (the bottom layer in the U-shaped architecture). 

In this work, the Retina U-Net was implemented to segment lung nodules. It sums up 6 layers in the contracting path (see Figure 2), 18 feature maps in the first layer and 576 in the deeper one. In the expansive path, on the other hand, the number of channels is half the ones in the first 4 layers, starting from 576, but then it is kept to 18 in the last 2 upper layers, consistently with the contracting path. 

#### 2.3.3. CADx Architecture and Development

**Figure 3:**
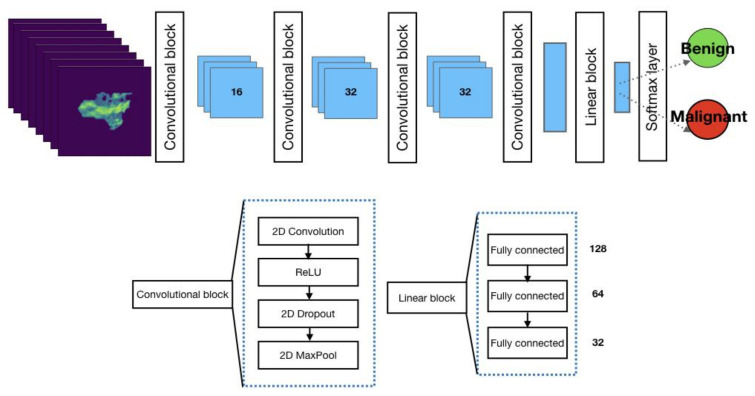
Architecture of the convolutional neural network used to classify lung nodules as benign or malignant. The number in each layer represents the number of output channels in that layer.

The neural network architecture adopted to classify lung nodules is a convolutional neural network (CNN) adapted from [15] (Figure 3). 

CNN consists of several layers responsible for feature extraction steps (four convolutional blocks) and classification (three fully connected layers and a SoftMax layer). The SoftMax function returns probability values for a given lung lesion, which is then classified as benign or malignant.

In Figure 3, the CNN layers are grouped into three blocks: the convolutional block, linear block, and SoftMax layer. 

The convolution block consists of a convolutional layer, ReLU (rectified linear unit), and 2D dropout. Unlike FCNN, CNN does not account for an expanding path because it is not designed to return full-resolution images; its output labels are related to the classification task. As noticeable in Figure 3, a stack of convolution blocks allows for down-sampling of the input image (CT scan) into feature maps that are subsequently ingested by a linear block. The latter consists of fully connected layers paramount to the classification task and ingests high-level features out of down-sampled feature maps from the previous layers. The last layer is characterized by the SoftMax function returning probability values for the input belonging to the category of interest. 

Training was performed using an equally balanced cross-entropy loss and Adam optimizer. Each series was preprocessed to extract the pixels belonging to lung nodules; indeed, the series was multiplied by the binary segmentation of each nodule. 

As a result, any pixel not belonging to lung nodules is considered a background pixel. In the inference phase, the binary mask of each nodule is the result of the segmentation network described in the previous section, followed by the CNN. 

The input volumes are centrally cropped around the lesion to a target size of eight slices, with a 100 × 100-pixel mask. During training, image augmentation is performed by applying random rotations, flipping, and brightness variation. The latter step is to increase the size of the training set to prevent the output model from being prone to overfitting. 

As can be noticed in Figure 3 the latest layer from the network stack is a SoftMax function, which is responsible for returning probability values. The likelihood value is then adopted to extract the classification target, which is the network output. 

The following section focuses on the system infrastructure and depicts the healthcare scenario we adopted in this study. 

## 3. System Infrastructure

**Figure 4:**
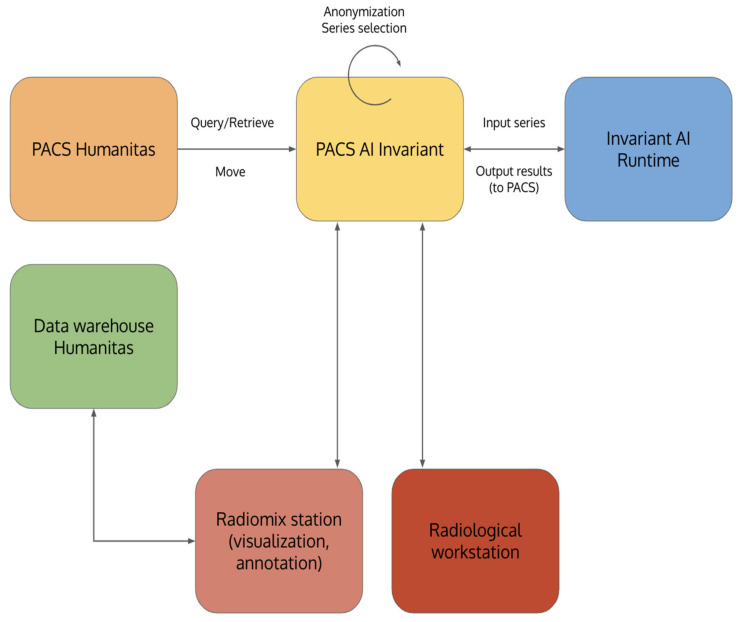
System infrastructure components: PACS Humanitas; PACS AI Invariant, Invariant AI Runtime; Data Warehouse; Radiomix Station, Radiological Workstation.

DICOM series identified from the institutional PACS as chest CT scan acquired and stored according to good clinical practice were downloaded and retrieved from the PACS AI Invariant. Data were anonymously stored in this layer to address privacy requirements compliance. Each series retrieved from the PACS AI Invariant was added daily to a DICOM series queue preprocessed in a cascade by the neural networks previously described. The Invariant AI Runtime module (Figure 4) was used to run the models. The results were then re-transferred to PACS AI Invariant to be processed, consulted, and envisaged on radiological workstations. The model results and manual annotations performed using the 3D-slicer plugin were stored in PACS AI Invariant and a data warehouse.

## 4. Metrics 

The detection rate, accuracy, specificity, and sensitivity were computed to evaluate the performance of the CADs and the CADx, respectively. Specifically, the “ground truth” for the CADs was the segmentation performed by the imagers (complete concordance between the imagers). The detection rate was calculated as the number of nodules correctly identified by the CADe and the total number of nodules segmented by the imagers. The Dice score was calculated to compare CADe’s and imager’s segmentation. The final diagnosis (radiological follow-up or pathology) represented the reference standard to evaluate CADx’s performance. Accordingly, each CADx prediction was classified as true positive, true negative, false positive, or false negative. The confidence analysis was used to evaluate the distribution of the probability values of each predicted nodule to belong to its class. The abovementioned metrics were calculated for training, validation, and test sets.

## 5. Results

As mentioned above, CADe and CADx were independently developed, trained, and tested. The results of CADx (i.e., classification) were not related to the CADe’s prediction (i.e., segmentation). We reported the results of the performance obtained in the test set. 

### 5.1. CADe 

**Figure 5:**
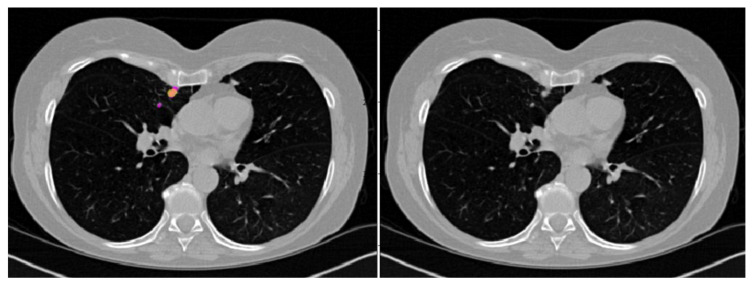
Example of a nodule close to pleura in the right lung correctly predicted by the CADe and of a small nodule, near to the previous one, missed by the CADe. Left panel: axial CT slice with prediction (yellow) and/or mask (pink); right panel: original CT image.

CADe correctly identified 96/123 nodules (78%) and missed 27/123 nodules. Specifically, 90/104 and 6/19 nodules of the ICH_s1 and ICH_s2 datasets, respectively, were detected correctly. Failures were relayed mainly on ground glass opacity (*n* = 6) and very small or very large nodules close to vessels, pleura (Figure 5), and/or mediastinum (*n* = 6, *n* = 9, and *n* = 4, respectively). 

An average of 10.84 nodules per series were falsely identified. The number of false positives was reduced to 6.5 nodules per series when excluding nodules smaller than 3 mm.

### 5.2. CADx

**Figure 6:**
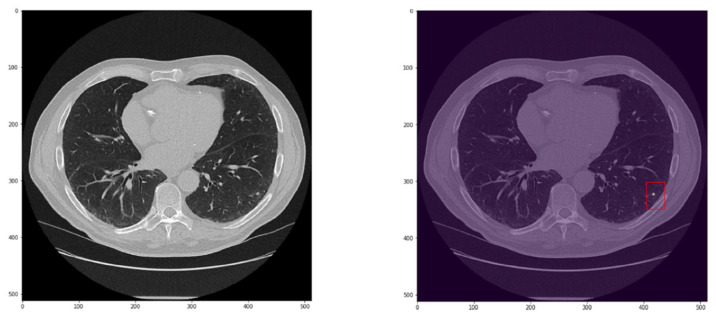
Example of a solid nodule of 6.3 mm (inside the red box) wrongly predicted as benign by the CADx.

**Figure 7:**
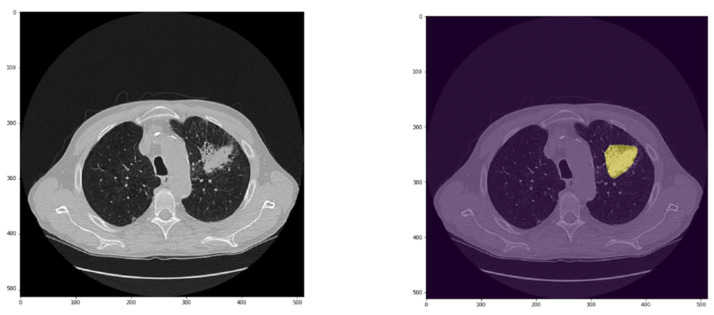
Example of a lesion of 55 mm (in yellow) wrongly predicted as malignant by the CADx.

CADx correctly classified 109/136 nodules (43 true negatives and 66 true positives). The CADx failed in classifying 27 nodules (11 false negatives and 16 false positives, Figure 6 and Figure 7). The size of nodules wrongly classified was between 3 and 6 mm in 7/27 cases (6/7 solid and all falsely classified as benign), greater than 6 mm but smaller than 8 mm in 5/27 cases (3/5 solid and 2/5 falsely classified as malignant), between 8 and 10 mm in 2/27 cases (both ground glass opacity resulted false positive), bigger than 10 mm but less than 15 mm in 2/27 cases (both solid, one resulted in a false negative and one resulted in a false positive), between 15 and 25 mm in 9/27 cases, and greater than 25 mm in the remaining 2/27 cases. Specifically, false negative nodules were small nodules with a median size of 4.85 mm (range 3–11.3 mm) and solid in the majority of the cases (8/11). Considering only solid nodules, the median size of lesions falsely classified as negative was 4.7 mm (range 3–11.2 mm). Three round glass opacities (median size of 7 mm, range 3.3–7) were wrongly classified as benign. False positive nodules were quite big nodules with a median size of 20 mm (range 7.2–55 mm). Nodules wrongly classified as malignant were mainly solid (10/16) with a median size of 22 mm (range 7.2–55 mm). Considering only this class (i.e., solid nodules resulted in false positives), a consistent number of nodules (7/10) were bigger than 15 mm. Other false positive results accounted for ground glass opacity (*n* = 3/16) and part-solid nodules (3/16) with a median size of 10 mm (range 9–15 mm) and 23 mm (range 20–23 mm), respectively. 

Our CADx system achieved an 80% accuracy rate. The sensitivity and specificity rates were equal to 85.7% and 73%, respectively.

**Figure 8:**
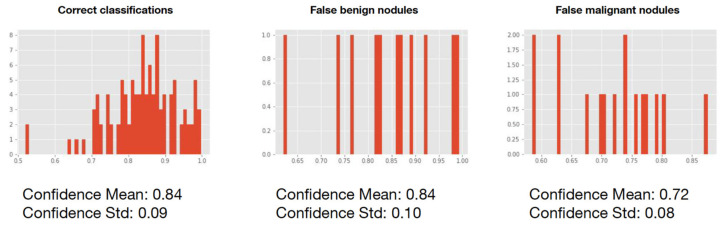
Confidence mean and standard deviation for correct classifications, false benign nodules and false malignant nodules.

The graphs in Figure 8 show the probability of each predicted nodule belonging to its class being similar for correctly classified lesions and nodules misclassified as benign (mean = 0.84 and standard deviation = 0.09 and mean = 0.84 and standard deviation = 0.10, respectively). In contrast, the confidence mean of the CAD in incorrectly predicted malignant lung lesions was lower (mean = 0.72 and standard deviation = 0.08, Figure 7).

## 6. Discussion

We developed an “intelligent agent” to detect and non-invasively characterize lung lesions using any type of CT scan. Big nodules detected incidentally are typically not a challenge for clinicians since the size and radiological characteristics rarely leave room for doubt. In contrast, nodules of less than 1 cm may be uncertain and difficult to characterize. In this setting, based on patient risk assessment (low versus high), number (solitary versus multiple), pattern (solid, part-solid, and ground glass), and the size of the nodule, radiological follow-up, [18F]FDG PET/CT and biopsy are recommended [3]. However, these actions might be not feasible and/or can result in inconclusive results. Therefore, a tool able to correctly classify at least small nodule (3–8 mm) as benign or malignant is actually an unmet clinical need. As mentioned, our CADe missed some nodules (22%), mainly ground glass opacity or nodules close to vessels, pleura, or mediastinum. Notably, all nodules of 15 mm or greater were wrongly classified as false positives, while the majority of nodules smaller than 10 mm (77%) resulted in false negatives. Collectively, our CADx was more sensitive than specific and wrongly classified 20% of nodules (8% as false negatives and 12% as false positives).

Performant algorithms capable of detecting lung lesions and discriminating benign from malignant nodules with great accuracy have been described [4,6]. Our CADe and CADx exhibited lower accuracy for both detection and classification (78% and 80%, respectively) tasks than those achieved by the algorithms reported in the literature (up to 95% [6] and 96% [4], respectively). Our CADe missed some ground glass opacities and close-to-vessel nodules, pleura, and/or mediastinum. Similar failures have been reported for deep learning-based algorithms in the literature [16]. Nonetheless, our CADs-CADx benefitted in some respects. Firstly, they were developed and tested using a local dataset from real-scenario data including different types of CT images (co-registered CT from PET/CT = 23%, biopsy-guiding CT scans = 23%, low-dose CT = 27%, and fully diagnostic CT = 27%). The performance achieved in highly selected and homogeneous datasets may lead to overestimated model reliability. Therefore, continuous “real-world” re-validation is necessary for clinical implementation of DL-based tools.

Secondly, our dataset consists of well-balanced classes of benign and malignant nodules (47% and 53%, respectively). Thirdly, the final diagnosis does not rely on subjective interpretative criteria to assess malignancy risks.

Conversely, we used pathology or a rigorous radiological criterion to determine whether a nodule was benign or malignant (approximately 60% and 40% of cases, respectively). Several deep-learning-based algorithms developed to detect and classify lung nodules relied on public datasets consisting of low-dose CT images collected within lung screening programs [4,6], which dealt with a low prevalence of relatively small nodules. Many publicly available databases see the risk of malignancy assessment by expert imagers as the “ground truth” [17,18,19]. Nonetheless, the latter has been recently shown to affect CADx’s reliability and performance [16]. Moreover, in many experiments, malignant nodules accounted for approximately one-third of the total number of nodules [20,21,22], potentially causing overfitting and ultimately affecting the model’s reliability. Lastly, malignancy in our datasets comprised primary lung tumors and lung metastases (81% and 19%, respectively). The pattern recognition out of CNN has shown similarities to typical image-feature-based learning [23]. Still, different imaging-based features in primary lung tumors and metastases have been reported [24], suggesting specific histology-based descriptors. 

On one hand, all these factors, although theoretically positive, generated a widely heterogenous dataset which was analyzed using the gold standard as a reference, which possibly explains why our tool was less performant than those reported in the literature. On the other hand, with the dataset being more heterogeneous, it positively impacted the overfitting and the generalizability of the CADs-CADx in the “real world”. Therefore, we can realistically consider our CADx as a tool—albeit to be further improved—for a “virtual biopsy”. It could result in several worthwhile circumstances, including, among others, lung nodules of undetermined significance. Giles et al. [25] reported that lung nodules of unknown significance were malignant in 86% of cases. Notably, in this series of 500 surgically treated patients, the percentage of lung metastases was not negligible concerning the total number of malignant lesions (22% metastases versus 78% primary lung tumors) [25], thus underlying the potential additional value of our CADx. Moreover, synchronous and metachronous tumors incidentally detected during staging or follow-up examinations have increased [26], making it imperative to exclude malignancy in a patient with a newly diagnosed lung nodule and a history of cancer. 

Despite the abovementioned positive aspects, this study also presented some limitations. Firstly, the CADs-CADx were independently developed, and the presented results refer to the detection and classification tasks separately. The next step will be to test the end-to-end tool on independent data. Furthermore, the algorithms’ architectures used for the CADs-CADx were modified from pre-existing neural networks. That is common for real scenario-oriented deep learning, with fewer methodological and theoretical contributions than new, application-oriented results; the novelty is often represented by the employment of pre-existing deep learning techniques applied in new scenarios and research fields through context-based modifications. 

The consideration above paves the way to a crucial point in the reliability of so-called supervised deep learning for some specific tasks. Two main questions arise from our experimental results: Can CNNs and FCNNs be considered as reliable tools for CADe and CADx? Is the supervised learning paradigm gradually going to be left behind in favor of semi-self-supervised deep learning architectures? 

The paradigm adopted might not be the most suitable for a scenario with several constraints: images with different spatial resolutions and various sensors’ fingerprints. The latest progress in AI sees new architectures reliant on self-supervised learning, which move toward AGI (artificial general intelligence) capable of inferring hidden properties from input data to be fine-tuned over a specific target with only a limited number of annotated samples. The results bring up some other aspects that deserve further investigation. For example, our experimental campaign ran essential data augmentation to prevent lung lesion shape distortion. Nonetheless, more advanced augmentation techniques based on generative deep learning, such as GANs (generative adversarial networks), appear to be promising to provide datasets with many more samples to be re-utilized for training purposes.

All that said, as for other domains of image patter recognition (e.g., animal photos) [27], we are convinced that sophisticated algorithms are insufficient in the setting of “real-world” data, and a huge number of observations (A million? A billion?) are needed to reach satisfactory results in terms of sensitivity and specificity. Moreover, we should keep in mind that our final goal is to develop a tool able to reach 100% accuracy, since even only one misclassified case is a misdiagnosed patient.

## 7. Conclusions

We have presented a specific case study on the detection and classification of lung lesions on CT scans to test the effectiveness of two of the most popular deep learning architectures, FCNN and CNN. To this end, we employed data from datasets with different features and specs. The first one was the LUNA 16 Challenge dataset; the second one consisted of images locally acquired and labelled. Furthermore, CT scans were acquired with different scanners making the case study close to real scenarios with the probability of unknown information about the sensors generating the images undergoing CADe and CADx checks. The experimental campaign confirmed the promise of these approaches in automated lung nodule assessment on CT, alongside with some drawbacks of the supervised learning paradigm employed in networks such as CNN and Retina U-Net in real-world clinical scenarios, with CT scans from different devices with different sensors’ fingerprints. Collectively, we proved that these tools, although promising, are not “mature” enough to successfully analyze “real-world” data and to be finally implemented in clinical practice. 

## Figures and Tables

**Table 1:** Lung abnormalities annotated within the LUNA challenge dataset.

Nodule ≥ 3 mm	Complete region of interest (ROI) boundary (>1 point) Nodule characteristics (e.g., roundness, sharpness of the margin, internal structure, etc.)
Nodule < 3 mm	The approximate centroid of the nodule No characteristics
Non nodule > 3 mm	The approximate centroid of the nodule No characteristics

**Table 2:** CT series datasets used for the CADs’ development.

Dataset	Training	Validation	Test	Total
LUNA	603	202	-	805
ICH_s1	764	191	234	1189
ICH_s2	54	19	19	92
Total	1421	412	253	2086

**Table 3:** The number of lung nodules included in each dataset used for the CADx development.

Final Diagnosis	Training	Validation	Test	Total
Benign nodule	381	192	59	632
Malignant nodule	439	198	77	714
Total	820	390	136	1346

## Data Availability

Four local and private datasets, namely, ICH_s1, ICH_s2, ICH_x1, and ICH_x2, were collected and labelled in IRCCS Humanitas. They are stored in the institutional archive; they are available from the corresponding author on reasonable request. The LUNA Challenge dataset is publicly available [9].
